# Aflatoxin M1 Concentrations, Adulterants, Microbial Loads, and Physicochemical Properties of Raw Milk Collected From Nekemte City, Ethiopia

**DOI:** 10.1155/2024/3796985

**Published:** 2024-09-07

**Authors:** Leila Nasir, Habtamu Fekadu Gemede

**Affiliations:** Department of Food Technology and Process Engineering Wollega University, P.O. Box 395, Nekemte, Ethiopia

**Keywords:** adulterants, aflatoxin M1, cow's milk, Nekemte, physicochemical

## Abstract

Milk is an essential part of the human diet and is a nutrient-rich food that improves nutrition and food security. The aim of this study was to determine the presence and concentration of aflatoxin M1 (AFM1), adulterants, microbial loads, and physicochemical properties of raw cow's milk (CM) in Nekemte City, Ethiopia. A total of 12 samples of fresh CM were purposefully collected from four kebeles in the city (Bake Jama, Burka Jato, Cheleleki, and Bakanisa Kese) based on the potential of each milk production and distributor site. The AFM1 concentration was determined by high-performance liquid chromatography (HPLC) with a Sigma-Aldrich standard (St. Louis, MO, USA). The concentrations of AFM1 in Bake Jama, Burka Jato, Cheleleki, and Bakanisa Kese were found to be 0.01–0.03 g/L, 0.31–0.35 g/L, 0.19–0.21 g/L, and 0.04–0.07 g/L, respectively. The concentrations of AFM1 in the present study varied significantly (*p* < 0.05) and ranged from 0.01 g/L to 0.35 g/L. These results show that of the 12 samples tested, all were positive for AFM1 and contaminated to varying degrees. The results of this study also revealed that the concentration of AFM1 in 7 (58%) of the 12 milk samples was above the European Union's (EU) maximum tolerance limit (0.05 g/L). The present study also revealed that of the investigated adulterants, only the addition of water had positive effects on three milk samples, while the remaining adulterants were not detected in any of the milk samples. The total bacterial count (TBC) and total coliform count (TCC) were significantly (*p* < 0.05) different and ranged from 5.53 to 6.82 log_10_cfumL^−1^ and from 4.21 to 4.74 log_10_cfumL^−1^, respectively. The physicochemical properties of the milk samples in the present study were significantly (*p* < 0.05) different and ranged from 2.8% to 5.75% fat, 7.03% to 9.75% solid-not-fat (SNF), 2.35% to 3.61% protein, 3.33% to 5.15% lactose, 11.54% to 13.69% total solid, 0.16% to 0.18% titratable acid, 26.7 to 32.1°C, 6.35 to 6.55 pH, and 1.027 to 1.030 specific gravity. The physicochemical parameters of the raw milk in the study area met the required quality standards. Hence, further studies are required to determine the extent of the problem and the factors associated with high levels of AFM1 in raw milk in the study areas, including the detection of aflatoxin B1 (AFB1) in animal feed.

## 1. Introduction

Humans have consumed milk as one of the most popular and healthy foods since prehistoric times. Milk has many nutritional enrichment benefits [1]. Mycotoxins are toxic secondary metabolites [2–4] that are naturally produced by molds such as *Aspergillus*, *Fusarium*, and *Penicillium* species [5, 6]. There are approximately six main types of aflatoxins (AFs) based on their ultraviolet fluorescence. Aflatoxin B1 (AFB1) and aflatoxin B2 (AFB2) emit blue fluorescence, whereas aflatoxin G1 (AFG1) and aflatoxin G2 (AFG2) emit green fluorescence. 4-Hydrated AFB1 and AFB2 are converted to aflatoxin M1 (AFM1) and aflatoxin M2 (AFM2), respectively [7].

AFM1 refers to a milk toxin present in aflatoxin-contaminated animal feed. AFB1 is the most toxic and widespread [6, 8]. It has potent teratogenic, mutagenic, and carcinogenic effects [9, 10]. A direct relationship was found between the amount of AFB1 consumed and the amount of AFM1 secreted in milk. AFM1 is released into milk in amounts ranging from 0.3% to 6.2% of the AFB1 ingested by animals [7]. AFM1 is resistant to thermal inactivation, pasteurization, autoclaving, and other varieties of food processing procedures [11]. Thus, to produce high-quality milk, it is essential to keep feeds free from contamination by AFB1 [12].

AFM1 is responsible for hepatotoxicity, cancer, nutritional interference, immune suppression, and teratogenesis in humans [13]. Moreover, exposure to AFs can cause growth impairment and immune suppression [14]. For these reasons, the last Commission Regulation of the European Union (EU) no. 165/2010 considered stringent parameters for AF regulation. A concentration of 0.05 mg/L was used for AFM1 in milk. The US Food and Drug Administration (USFDA) set the level of AFM1 in milk to 0.5 mg/L [14]. The European Commission established the maximum permitted level for AFM1 to be 0.025 *μ*g/kg for infant formula and 0.05 *μ*g/kg for raw milk [13]. Ethiopia uses the regulatory limits set by the European Commission (0.05 *μ*g/kg for raw milk). The USFDA has set the AFM1 concentration in dairy products at 0.5 *μ*g/L [14].

In addition, AFM1 adulterants are the major factors that affect the quality of milk [14]. For economic purposes, adulteration substitutes large substances with low prices at high prices. For example, the chloride in milk interrupts the acid–base balance in the body as well as the pH of the blood [15]. Milk adulteration became a global concern after the 2008 outbreak of melamine-contaminated infant milk products in China [16]. One of the main factors affecting milk quality is the microbial load [17]. However, microbial loads indicate that milk is contaminated at different stages of processing [18]. Milk is a complete food because it is the main source of protein, fat, and minerals. The solid components of milk, mainly fats and proteins, make milk an important nutritional and economic asset [17, 19].

Several international studies have shown higher levels of AFM1 in milk [13] and AFM1 contamination in raw milk worldwide, and the concentrations of AFM1 exceeded the European Community and Codex Alimentarius regulatory limits [13]. The AFM1 contamination in milk and dairy products in different cities of Ethiopia [13, 14, 20] has also shown that milk is highly contaminated at different concentrations of AFM1 [14], which also differs from one place to another in the country, which may be related to the climatic conditions of each geographical area and differences in the feeding system of dairy cows [21, 22]. Patyal et al. [23] also reported that animal feed storage and leftover household cereals, longer feed storage duration, and feed storage quality were significantly associated with the presence of AFM1 in farm milk. However, there is little published information on the levels of AFM1 and the microbial load in fresh cow's milk (CM) in Nekemte City, Ethiopia. Therefore, this study aimed at evaluating the level of AFM1 and the microbial load of raw CM. In addition, the investigation evaluated the adulterants and physicochemical properties of the raw milk.

## 2. Methods

### 2.1. Description of the Study Site

Nekemte City is located west of the Oromia region. The word Nekemte was derived from the owner of the land, whose name was “Nekemte Gada Otaa,” who had lived for a long time. Administratively, it is divided into six subcities: Darge, Bake Jama, Burqa Jato, Bakanisa Kese, Cheleleki, and Keso. Nekemte has a latitude and longitude of 9°5′N 36°33′E/9.083°N 36.550°E and an elevation of 2,088 meters above sea level. The average temperature of the city is 17.6°C, and the average annual rainfall is 1988 mm.

### 2.2. Data Collection

A total of twelve raw CM (500 mL each) samples were collected from individual raw CM distributors of four kebeles (Bake Jama, Burka Jato, Cheleleki, and Bakanisa Kese). The specific distributors in each milk collection kebele were selected purposefully by considering the locations where the kebele was distributed and potential distributor individuals having more than 5 cows. The milk samples reached the distribution center within three hours and were collected in precleaned and dry polyethylene bottles (ASTM D2911/0.25 mm–0.89 mm) [47]. The polyethylene bottles were rinsed with the sample. The samples were collected from each distributor, kept in an ice box, and transported to the laboratory for further analysis.

### 2.3. AFM1 Analysis

The frozen samples were warmed in a water bath at 37°C for 30 min, and 25 mL of the prepared sample was added to a 50-mL Falcon tube and centrifuged at 4000 rpm for 5 min. Then, the solution was filtered through fast flow (541) filter paper attached to an immune-affinity column (EagleBio Immunoaffinity Columns, Cat. no. BTAFM311005), which was used to separate AFM1 from the mixture of the solution, and the filtrate was passed through an immune-affinity column at 1 drop/sec. Then, the immune-affinity column was washed with 5 ml of 10 M phosphate-buffered saline (PBS) three times and allowed to dry in air. AFM1 was eluted with 1 mL of methanol three times, filtered through a 0.45-*μ*m syringe filter into an amber autosampler, and transferred to a 2-mL vial for high-performance liquid chromatography (HPLC) analysis [16].

**Figure 1:**
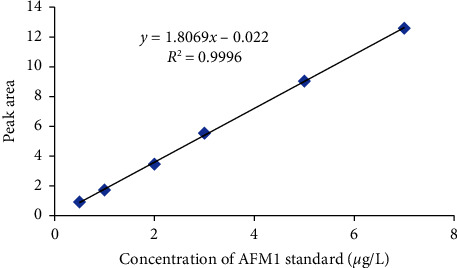
Calibration curves of standard AFM1.

Standard solutions were used for preparing the intermediate solution, and then, working standard solutions were prepared from the intermediate solution of AFM1. The calibration curve was plotted with six points for each standard (0.5 *μ*g/L, 1 *μ*g/L, 2 *μ*g/L, 3 *μ*g/L, 5 *μ*g/L, and 7 *μ*g/L) using the peak area against the concentration, and the calibration curve is given in Figure 1. The linearity of the HPLC-FLD system was determined by injecting different concentrations of standard, and the system was calibrated by using working solutions of AFM1 in the range of 0.5–7 *μ*g/L. The response (peak area) is given in Table 1.

The calibration curve of AFM1 was constructed by plotting the peak area of AFM1 versus the concentration of the working standard AFM1, as shown in Figure 1. The method was linear for AFM1 concentrations ranging from 0.5 to 7 *μ*g/L. From Figure 1, the equation describing the calibration curve was *Y* = 1.8069*X* − 0.022, where the *Y*-axis is the peak area and the *X*-axis is the concentration of AFM1 (*μ*g/L). The analyzed working solution gave excellent coefficient of regression values for AFM1. The coefficient of regression (*R*^2^) value was 0.9996, which is considered evidence of an acceptable fit of the data to the regression line.

### 2.4. Determination of Adulterants

Adulteration tests were conducted to detect common adulterants (water, starch, cane sugar, formalin, acidity, hydrogen peroxide, and common salt) in raw CM collected from Nekemte. Qualitative detection of adulterants in milk involves simple color-based chemical reactions via color charts [18].

### 2.5. Determination of the Microbiological Load

Microbiological load tests, such as total bacterial count (TBC), total coliform count (TCC), and yeast and mold counts, were performed to determine the microbial status of the milk samples. TBC (cfu/mL) was generated by following the method recommended by the American Public Health Association [4].

### 2.6. Determination of Physicochemical Properties

Analyses were performed at Nekemte (Dambeli Dairy Farming and Processing Factory laboratory) by a lactoscanner (serial no. SP-BJ-000883 made in Bulgaria) to determine the composition of milk (fat, solid-not-fat [SNF], protein, and lactose). The lactoscan was adjusted for CM sample analysis. Then, the CM sample was taken into a sample holder and placed under the sensors of the analyzer. The enter button was pressed to start the test. After 40 seconds, the reading result was taken for each parameter. The analyzer was rinsed with distilled water after each sample analysis. The percentage of total solids was calculated by the following equation: % total solids = % SNF + % fat, titratable acidity, pH value, and specific gravity [24, 25].

### 2.7. Statistical Analysis

The microbial load and physicochemical property data were analyzed by one-way analysis of variance (ANOVA). The microbial load counts were first converted to logarithmic values (log_10_), and these values were analyzed. Descriptive statistics such as frequency distributions and percentages were analyzed using the Statistical Package for the Social Sciences, version 23 (IBM SPSS Inc., Chicago, USA). Data obtained from laboratory analyses were analyzed by ANOVA to compare the mean values of the treatments using least significant difference (LSD) tests at a significance level of *p* < 0.05.

## 3. Results and Discussion

### 3.1. AFM1 Concentrations in Milk Samples

Table 1 shows the AFM1 concentrations of the analyzed samples from the study area. The AFM1 concentrations in the raw milk from Bake Jama, Burka Jato, Cheleleki, and Bakanisa Kese ranged from 0.01 to 0.03, 0.31 to 0.35, 0.19 to 0.21, and 0.03 to 0.07 *μ*g/L, respectively. The AFM1 concentrations in the raw milk from Bake Jama, Burka Jato, and Bakanisa Kese were significantly (*p* < 0.05) different, whereas there was no significant (*p* > 0.05) difference among the samples collected from Cheleleki. The AFM1 concentration in the study area ranged from 0.01 *μ*g/L (Bake Jama, S2) to 0.35 *μ*g/L (Burka Jato, S2). The samples were collected in the winter season, which is characterized by dryness and grazing food scarcity. In such cases, dairy farmers may rely on additional feed and feed their cows regardless of availability, with no regard for fungal contamination of the feed. This might be the reason for AFM1 contamination in the milk sample. Several methods have been introduced to reduce AFs in milk and dairy products. Some studies have shown that preventing crop contamination during the preharvest and postharvest stages is a useful strategy for eliminating fungal toxins. Other studies have proposed direct methods for reducing AFM1 in the milk [3, 4].

The mean AFM1 concentration ranged from 0.01 to 0.03 *μ*g/L (Bake Jama) in the raw milk sample, which was within the range reported by Amer and Ibrahim [33]. This result is also comparable to that of research on the raw milk from Sudan, which ranged from 0.0021 to 0.131 *μ*g/L [26]. The AFM1 concentration reported in this kebele was below the range of 0.028–4.98 *μ*g/g/L reported by Gizachew et al. [14]. This may be due to the use of grass as cow feed in the study area because the amount of AFM1 is lower in grass than in industry byproducts.

The mean AFM1 concentration in Burka Jato was comparable to that in the raw milk from Pakistan, which ranged from 0.01 to 0.76 *μ*g/L [27]. An investigation from urban centers in Kenya reported AFM1 levels of up to 0.68 *μ*g/L [28]. The AFM1 concentration ranged from 0.31 to 0.35 *μ*g/L in the present study, which was within the range of 0.028–4.98 *μ*g/L for AFM1 reported by Gizachew et al. [14]. However, the levels of AFM1 contamination found in the raw milk collected from Khartoum State in Sudan, within an average concentration of 2.07 *μ*g/L and a maximum concentration of 6.9 *μ*g/L [28], were higher than those found in this study. The milk sample from Burka Jato might have a lower AF concentration than the other milk samples because of the different feeding methods used. It has been reported that dairy cattle fed aflatoxin-contaminated feed produce contaminated milk [29].

As shown in Table 2, the mean AFM1 concentration in Cheleleki agreed with the results, showing that AFM1 contamination in the milk ranged from 0.02 to 0.31 *μ*g/L in Gurage Zone, Ethiopia [30]. These results were also within the range of 0.01–0.76 *μ*g/L AFM1 contamination in cow A [27]. The mean AFM1 concentration in Bakanisa Kese was comparable, ranging from 0.046 to 0.22 *μ*g/L for raw CM in the city of Injibara, Awi Zone, Amhara [31]. This sample might be the milk collected from pure pasture/grass-fed cows.

According to the oral interviews with distributors, the dairy farmers' cows were not completely grassed, and they were also fed dry grass, industry byproducts, waste from traditional beer, and other leftover food, all of which are favorable media for the growth and propagation of aflatoxin-producing fungi. Moreover, the AFM1 level in milk samples has been investigated in several countries. Studies from urban centers in Kenya have reported AFM1 levels of up to 0.68 *μ*g/L [28]. Compared to this result, the levels of AFM1 contamination found in the raw milk collected from Khartoum State in Sudan, with an average concentration of 2.07 *μ*g/L and a maximum concentration of 6.9 *μ*g/L, were greater [32].

The AFM1 contamination level was slightly higher than that in Egypt (0.05 *μ*g/L) [33]. However, the quality of milk in terms of the level of AFM1 in milk was lower than that in milk samples from Sudan [32]. The results of this study ranged from 0.01 to 0.35 *μ*g/L, which was within the range of 0.028–4.98 g/L reported for Ethiopian milk samples [14]. Additional investigations on small-scale dairy farms in Zimbabwe in 2016 revealed that the level of AFM1 contamination was in the 0.74–1.30 *μ*g/L range, with only 30% of the samples being positive. Makau et al. [34] reported that all milk samples from rural dairy systems had AFM1 contamination levels below the EU limits, ranging between 0 and 0.041 *μ*g/L. An investigation of raw milk samples from rural Punjab by Iqbal et al. [27] indicated that 52% of the tested samples (*n* = 48) were contaminated with AFM1, with a mean of 0.04 ± 0.034 *μ*g/L.

Table 3 shows the comparisons of AFM1 concentrations in milk samples in the present study with the EU limit (0.05 *μ*g/L). The AFM1 concentrations in the raw milk from Bake Jama ranged from 0.01 to 0.03 *μ*g/L, which was below the permissible limit of the EU/ES standard of 0.05 *μ*g/L and was below the EU/ES standard limit. However, the AFM1 concentrations in samples collected from Bake Jama and two samples from Bakanisa Kese were below the EU/ES standard limits. The AFM1 concentrations in all samples collected from Burka Jato ranged from 0.31 to 0.35 *μ*g/L and from Cheleleki ranged from 0.19 to 0.21 *μ*g/L, which were above the permissible limit of the EU/ES standard of 0.05 *μ*g/L. From the comparison results, all samples collected from Burka Jato, Cheleleki, and Bakanisa Kese exceeded the EU/ES standard limit (0.05 *μ*g/L).

The results indicated that the concentration of AFM1 in the present study was above the permissible standard limit in 7 (58%) of the 12 milk samples, whereas in 5 samples (42%) it was below the permissible standard; thus, this milk is considered safe for consumption. Aflatoxin exposure can cause cancer in many species; most states have recognized guidelines for AF levels in food and feed to limit exposure to this mycotoxin group [35]. The presence of AFM1 in milk is a serious concern globally due to its potent carcinogenicity [36].

Global hepatocellular carcinoma is the leading cause of cancer death [37]. Mycotoxins have drawn worldwide attention because of their significant impact on human health, animal productivity, and trade [38]. Aflatoxin poisoning is a serious food safety problem worldwide [39]. Hazardous human exposure to AFM1 through milk may occur [9]. The safety of food and feed for human and animal consumption should be the top priority with regard to the regulation of agricultural and food industries.

### 3.2. Analysis of Adulterants in Raw CM

Table 4 shows the results for the adulterants in the selected milk samples from the study area. In the present study, milk samples from the study area were examined for the presence of adulterants such as water, starch, cane sugar, formalin, acidity, hydrogen peroxide, and common salt. The adulterants were examined via simple color-based chemical reactions. The results showed that only one sample (S3) from Bake Jama and two samples (S1 and S3) from Cheleleki were positive for water adulteration. Two samples (S1 and S2) from Bake Jama, one sample (S2) from Cheleleki, and all samples from Burka Jato and Bakanisa Kese had negative responses to water adulteration. Of the twelve raw milk samples, three were found to be positive for water, which indicates the addition of water to the milk; none of the other milk samples showed the presence of adulterants.

Adulteration of milk with water is usually practiced in areas with access to the fresh milk market. From an economic point of view, milk is being adulterated. Gaining profit through the adulteration of milk is a major issue for developing countries, and it has adverse effects on the health of the population [40]. Chemical qualitative tests are the least expensive, most convenient, and most popular tests for detecting milk adulteration. These tests are performed with special skills [41].

Starch, cereal flour, or arrowroot is added to compensate for the density of the milk to prevent the detection of added water. Starch is also used as an adulterant, and if high amounts of starch are added to milk, this can cause diarrhea due to the effects of undigested starch in the colon. Cane sugar is added to increase the density to prevent the detection of extraneous water. Sodium chloride was added to determine the density (via a lactometer) of the watered milk [42].

Milk is rich in nutrients, and it is measured as a complete diet globally due to its nutritionally rich composition; therefore, milk and milk products are aimed at adulteration [43]. One of the most unreliable food processing methods is the use of milk adulterants, which have different adverse effects on human health, can cause serious diseases, and can reduce the nutritional value of milk [44]. The adulterants in milk are identified by changes in color due to the chemical reactions of different reagents. These reactions are valid only for small concentrations [45]. Generally, milk samples collected from Nekemte City were free from adulterants, except for Sample 3 (S3) from Bake Jama and one sample from Cheleleki (S1 and S3). Since the sample was free from chemical adulterants, it was safe for consumption.

### 3.3. Microbial Loads in the Raw CM

#### 3.3.1. TBC

This study revealed that the TBCs for all kebele species were not significantly (*p* > 0.05) different within the groups except for Burka Jato. The TBC ranged from 5.53 to 6.79 log_10_cfumL^−1^ (Table 5). The difference between the samples collected from Burka Jato might be due to the unsanitary handling of milking equipment. The mean TBCs in samples collected from Bake Jama, Burka Jato, Cheleleki, and Bakanisa Kese ranged from 6.31 to 6.48 log_10_cfumL^−1^, 5.53 to 6.62 log_10_cfumL^−1^, 6.65 to 6.82 log_10_cfumL^−1^, and 6.13 to 6.4 log_10_cfumL^−1^, respectively. High TBCs were detected in the milk sample collected at 6.75 log_10_cfumL^−1^ (Cheleleki), while low counts were detected in the milk sample collected at 6.24 ± 0.18 log_10_cfumL^−1^ (Burka Jato).

High TBCs might occur due to inadequate cooling of the milk, inadequate udder preparation methods, and unclean milking equipment. The results of this study were slightly lower than the results reported by Negash et al. [46], who reported a TBC of 7.08 ± 0.07 in the Mid-Rift Valley of Ethiopia. These values are also lower than those reported by Tola et al. [47], who reported 7.60 mL^−1^ in milk sampled from a small-scale producer in the Guto Wayu and Bila Sayo districts of East Wollega. High TBCs could indicate a diseased udder, unhygienic handling of milk, or unfavorable storage temperatures [48].

#### 3.3.2. TCC

TCCs did not significantly differ (*p* > 0.05) among the raw milk samples. The overall mean TCCs in samples collected from Bake Jama, Burka Jato, and Bakanisa ranged from 4.46 to 4.58 log_10_cfumL^−1^, 4.21 to 4.74 log_10_cfumL^−1^, 4.38 to 4.49 log_10_cfumL^−1^, and 4.37 to 4.4 log_10_cfumL^−1^, respectively.

The TCCs recorded in the milk samples collected from all study areas were comparable to the results of Tesfay et al. [49], who reported a count of 4.13 ± 0.76 log_10_cfumL^−1^ for the samples collected from dairy farms. The average coliform count of 4.43 log_10_cfumL^−1^ (Cheleleki) in the milk sample was similar to that reported by Tola et al. [47], who reported an average TCC of 4.46 mL^−1^ in milk samples collected from smallholder producers in the Guto Wayu and Bila Sayo districts of East Wollega.

The TCCs for all samples from the study area were lower than those reported by Debela [49], who reported that the mean TCCs of milk samples collected from markets and households were 6.455 and 6.192 log_10_cfu/mL, respectively. In this study, the coliform count of the raw milk collected from the study area was lower than the coliform count (4.94 ± 0.23 log_10_cfu/mL) of the milk samples collected from the Bahir Dar Zuria district. In this study, a high TCC (6.79 log_10_cfu/mL) was detected in the raw milk collected from Cheleleki.

The TCC results for the study did not meet the standard adopted by the EU limit, which was 102 cfu/mL. The mean TCC for all collected samples from the study area was comparable to the EAS maximum acceptable standard of 5.0 × 10^4^ log_10_cfumL^−1^ [50]. Total coliform organisms contaminate raw milk from unclean dirt, manure, hair dropping into milk during milking, udder washed with unclean water, dirty towels, and udder not dried before milking [51]. A similar phenomenon has also been described in different studies [52]. Thus, strengthening the extension services and training of milkers and distributors on improved milk handling practices are needed. Awareness raising and capacity building of various actors in the milk value chain is needed.

### 3.4. Physicochemical Properties

#### 3.4.1. Fat Content

The fat composition of the milk samples significantly (*p* < 0.05) differed. The minimum fat content was observed in the Bakanisa Kese sample (2.8 ± 0.18%), and the Bake Jama sample had a maximum fat content of 5.76 ± 0.18%, followed by the Burka Jato sample (5.43 ± 0.01%). This might be due to the cow breed and lactation stage. The high-fat composition obtained in this study was greater than that of crossbred cows in selected areas of Amhara and Oromia National Regional States, Ethiopia [53].

This could be because the fat content of milk varies from cow to cow and is affected by a number of factors: genetic breed, feed ratio, season, age of the cow, lactation stage, and contamination of the milk [54]. The minimum fat content in this study, 2.8% (Bakanisa Kese), was higher than that reported by Derese [57] in West Shoa Zone, Oromia Region, Ethiopia. This result agreed with that reported by Negash et al. (5.02%) [46].

The fat content reported in this study (5.76%) was high compared to the minimum quality standard value of the Ethiopian (ES) diet (3.50%) [55]. According to the FDA, the minimum fat content of whole milk is 3.25% [54]. This difference in percent fat content could be attributed to differences in cow breeds, feeding practices, and animal management practices. In this study, all the samples met the FDA requirements. Milk fat is the most desirable and important nutrient available in milk. The FDA [56] recommended no less than 3.25% milk fat for raw whole milk.

#### 3.4.2. SNF Content

The SNF composition of the milk samples significantly (*p* < 0.05) differed. The results range from 7.03% to 9.75% for S3 (Bake Jama) and S2 (Cheleleki), respectively. The SNF content reported in this study (9.75%) (Cheleleki) was comparable to that reported by Bekele et al. in Sebeta (8.93 ± 0.22) [58] and EU quality standards (8.25%) [55]. Similar results in Cheleleki were also described by Dehinenet et al. [53] for the solid fat content (8.88 ± 0.83) of milk samples. Additionally, the solid rather than fat content of milk for all kebeles was related to that of the control milk (8.42%) [55]. The high solid fat content (9.75%) obtained in this study was comparable to the 8.44% solid fat content reported by Dehinenet et al. [53]. This might be due to the lactation stage, age and health of the cows, feeding regime, and wholeness of milking. The results reported in this study were lower than the mean percentage of solid fat, not fat, 13.0%, in Sululta [59]. The reason could be the feeding and the lactation stage because as the lactation stage increases, the SNF decreases.

#### 3.4.3. Protein Content

The protein compositions of the milk samples were significantly (*p* < 0.05) different. The protein content of the raw milk samples ranged from 2.35% to 3.61%, as shown in Table 6. A low protein content (2.35%) was detected in the Bakanisa Kese raw milk sample (S3), whereas a high protein content (3.61%) was detected in the Bake Jama raw milk sample (S1). These variations could be due to differences in the milk samples from cow breeds and their feeding type. There was no significant difference between S1 and S2 from Bake Jama, while there were significant differences among all the other samples within and between the study areas. Tesfay et al. [56] reported 3.42% protein in the milk samples from Dire Dawa City, which was comparable with the results of this study.

The results revealed that a high protein content was detected in the sample from Bake Jama (3.61%), which was relatively similar to that in the study of raw milk sold at Hosanna, Ethiopia (3.40%), while a minimum protein content was detected in the sample from Bakanisa Kese (2.35%), which was close to the minimum result (2.75%) reported in Hosanna, Ethiopia, reported by Sebho and Meskel [60]. The maximum protein content (3.61%) in the present study was significantly greater than the average protein content (3.12%) in raw milk reported by Dehinenet et al. [53]. The protein content in this study (3.01%) was lower than that reported by Derese (3.61%) [57].

The low protein content in this study (2.35%) is consistent with the standard limit set by the EU (2.73%). The present study results (3.01%) slightly agreed with the minimum quality standard value of Ethiopia (3.20%) [55]. The protein content obtained in this study fulfilled the criteria developed by the FDA for consumers and agreed with EU standards. The protein content of milk might depend on the feed, cow breed, season, and lactation stage. Therefore, the protein content of the milk collected from the area agreed with the standards and with that of some other study areas.

#### 3.4.4. Lactose Content

The lactose content of the milk obtained from the study area was significantly different (*p* < 0.05). The lactose content of the raw milk samples ranged from 3.33% to 5.15%, as shown in Table 6. A high lactose content was obtained for S1 (Cheleleki), and a low lactose content was observed for S3 (Bakanisa Kese). This high percentage (5.15%) was related to the lactose content reported in the Sebeta area (4.91 ± 0.12) [58]. This finding was also comparable to the result reported by Bekele et al. (5.26%) [58].

Bacteria might utilize the lactose present in milk; thus, the lactose content of the milk could decrease. The lactose content also depended on the lactation stage; as the lactation stage increased, the lactose content decreased significantly. The low lactose content observed in milk collected from Bakanisa Kese might be related to the relationship between bacterial loads and lactose because as microbial loads increase, the lactose content decreases. The results of this study strongly agree with the lactose content set by EU standards (4.2%).

#### 3.4.5. Total Solid Content

The total solid content of the milk obtained from the study area significantly differed (*p* < 0.05). The total solid content of the raw milk samples in this study ranged from 11.54% to 13.69%. The maximum percentage (13.69%) was observed for S2, and the minimum percentage of S3 (11.54%) was observed for Burka Jato and Bakanisa Kese. The total solid content observed for Bakanisa Kese (11.54%) was lower than that observed for Burka Jato (13.69%), followed by the Bake Jama sample (13.15%). The maximum result was lower than that reported by Tola et al. (14.31%) [47]. The total solid content determined in this study (13.69%) was similar to that reported by Bekele et al. (13.48%) [58].

Additionally, the lowest total solid (11.54%) content obtained in this study was low, compared to the mean of 13.07% reported by Derese [57] and 13.15% for crossbred CM reported by Gizachew et al. [14]. In this sample, the percentage of total solids (13.69%) was comparable to 12.8% [55]. The total solid contents of samples of this study from Bakanisa Kese were less than those reported in the Ethiopian and EU standards (12.8% and 12.5%, respectively) [55]. According to the EU, recognized quality standards for the total solid content of CM should not be less than 12.5% [61]. The obtained total solid content was comparable to the quality standards.

#### 3.4.6. Titratable Acidity

The titratable acidity of the samples obtained from the study area was not significantly different (*p* > 0.05). Titratable acidity is a milk quality indicator that reflects the freshness, bacterial activity, and flavor of the milk. The titratable acidity ranged from 0.16% to 0.18% lactic acid. In this investigation, raw milk samples from Burka Jato and Bakanisa Kese had a low lactic acid content of 0.16%, whereas those from Cheleleki and Bake Jama had a maximum lactic acid content of 0.18%. These results were in the range of lactic acid values (0.17–0.22%) reported in Bangladesh [61]. The high acidity of the milk collected from Cheleleki and Bake Jama could be due to rapid bacterial spread and reproduction during the transport of the milk from the farm to the distribution center. Table 5 shows the microbial loads in the milk samples; the maximum bacterial loads were observed for Cheleleki, followed by Bake Jama. This could be due to bacterial growth and increased acidity of the milk.

#### 3.4.7. pH Value

The mean pH values of the samples were significantly (*p* < 0.05) different. A high pH was obtained for S1 (Bake Jama), and a low pH was observed for S3 (Baka Jama). The overall mean pH of the study area was 6.36 ± 0.14. The average pH of the milk collected from Bake Jama goats was 6.35, which was below the standard limit set by Eshetu et al. [61] of 6.6–6.8 for normal milk samples. The milk from Bakanisa Kese has a pH of 6.55, which was comparable to the range set by Mitiku et al. [61]. The pH of the milk samples from the Bakanisa Kese was significantly greater than that of the other milk samples.

The low pH of the milk collected from Cheleleki may be due to the production of acid resulting from bacterial growth and the increase in the milk samples. In milk samples kept at room temperature, the bacteria multiply, utilize lactose, and convert into lactic acid, thus increasing the acidity and decreasing the pH. Milk samples from the Bakanisa Kese were somewhat under the required standard; however, those from the Bake Jama area were acidic.

#### 3.4.8. Specific Gravity

The specific gravity did not significantly differ among the study areas (*p* > 0.05). For common whole CM, the specific gravity ranges from 1.028 to 1.036 kg/L based on the East African Community standard [50]. Having a specific gravity below the revealed level implies that there was adulteration of milk with water, which contributes to the production of poor-quality milk [62]. Raw milk from Bake Jama and Cheleleki has a lower specific gravity than that of the East African Community standard. Overall, 33% of the 12 samples had a specific gravity below 1.028 kg/L. These results indicate that water was added.

The disposal of milk with water might lead to chemical or microbial health hazards and reduce the nutritional and processing value, palatability, and marketing value of the milk [62]. The specific gravity of the samples from Burka Jato and Bakanisa Kese was in the range of the recommended level of the East African Community standard. The specific gravity of the raw milk from Bakanisa Kese was comparable to the specific gravity of the raw milk studied in the Guto Wayu and Bila Sayo districts of East Wollega. Water adulteration decreases the specific gravity of milk [63].

Generally, the composition and properties of raw CM vary from kebele to kebele. This could be due to differences in cow breed, lactation stage, type of feed, and health status. The overall mean chemical composition of the raw CM from the study area was related to both the Ethiopian and EU quality standards. The physicochemical properties of the milk from the study area include a high fat content, a lack of solid fat, and a total solid composition. Therefore, the raw milk collected from the study area had good nutritional quality.

## 4. Conclusion

This study aimed at evaluating the presence and concentration of AFM1, adulterants, microbial loads, and physicochemical properties of raw CM in Nekemte City, Ethiopia. The results of this study showed that the AFM1 concentration obtained from 12 potential milk distributors ranged from 0.01 *μ*g/L to 0.35 *μ*g/L. The results of this study also revealed that, of the 12 samples, 7 (58%) milk samples had levels that were higher than the acceptance limit determined by the EU (0.05 *μ*g/L). In the present study, water was the only adulteration present in three milk samples. A higher TBC was recorded in the milk collected from the Cheleleki raw milk sample (6.75 ± 0.18), while it was lower in the milk collected from Burka Jato (6.24 ± 0.18). TCCs were not significantly different. Yeast and molds were not detected. The microbial counts did not meet the international acceptable limits. Generally, the results showed that the quality of milk obtained from the study areas was low. Compared with those of the other kebeles, the physicochemical properties of milk from Bake Jama include a high percentage of fat, protein, SNF, and total solids. The overall mean milk composition in the study area was similar within the range of the Ethiopian standard value.

## Figures and Tables

**Table 1:** Calibration table of standard AFM1.

**Standard AFM1 (** ***μ*g/L)**	**0.5**	**1**	**2**	**3**	**5**	**7**
Peak area	0.929	1.734	3.466	5.542	9.039	12.586

**Table 2:** Aflatoxin M1 concentration in milk samples from the study area.

**Sample code**	**Aflatoxin M1 in raw cow milk (** ***μ*g/L**)			
	**Bake Jama**	**Burka Jato**	**Cheleleki**	**Bakanisa Kese**
S1	0.02 ± 0.01	0.31 ± 0.12	0.2 ± 0.02	0.04 ± 0.02
S2	0.01 ± 0.02	0.35 ± 0.13	0.19 ± 0.01	0.07 ± 0.02
S3	0.03 ± 0.02	0.33 ± 0.13	0.21 ± 0.02	0.03 ± 0.01
Mean	0.02 ± 0.01	0.33 ± 0.02	0.20 ± 0.01	0.05 ± 0.02

Abbreviations: S1, Sample 1; S2, Sample 2; S3, Sample 3.

**Table 3:** Comparisons of the AFM1 concentration (*μ*g/L) in milk samples with that of standards.

**Sample code**	**Aflatoxin M1 in raw cow milk (** ***μ*g/L)**			
	**Bake Jama**	**Burka Jato**	**Cheleleki**	**Bakanisa Kese**
S1	0.02 ± 0.01	0.31 ± 0.12	0.2 ± 0.02	0.04 ± 0.02
S2	0.01 ± 0.02	0.35 ± 0.13	0.19 ± 0.01	0.07 ± 0.02
S3	0.03 ± 0.02	0.33 ± 0.13	0.21 ± 0.02	0.03 ± 0.01
EU/ES standard	0.05			

Abbreviations: S1, Sample 1; S2, Sample 2; S3, Sample 3.

**Table 4:** Results of adulterants in the selected milk samples from the study area.

**Parameter**	**Milk sample**												**Total**	
	**Bake Jama**			**Burka Jato**			**Cheleleki**			**Bakanisa Kese**				
	**S1**	**S2**	**S3**	**S1**	**S2**	**S3**	**S1**	**S2**	**S3**	**S1**	**S2**	**S3**	**+ve**	**−ve**
Water	−	−	+	−	−	−	+	−	+	−	−	−	3	9
Starch	−	−	−	−	−	−	−	−	−	−	−	−	−	−
Cane sugar	−	−	−	−	−	−	−	−	−	−	−	−	−	−
Formalin	−	−	−	−	−	−	−	−	−	−	−	−	−	−
Hydrogen peroxide	−	−	−	−	−	−	−	−	−	−	−	−	−	−
Acid	−	−	−	−	−	−	−	−	−	−	−	−	−	−
Common salt	−	−	−	−	−	−	−	−	−	−	−	−	−	−

Abbreviations: +ve, presence of adulterants; −ve, absence of adulterants; S1, Sample 1; S2, Sample 2; S3, Sample 3.

**Table 5:** Total microbial counts of the raw milk samples (mean + std).

** **	**Sample code**	**Bake Jama**	**Burka Jato**	**Cheleleki**	**Bakanisa Kese**
TBC (log_10_cfumL^−1^)	S1	6.48 ± 0.18^a^	6.62 ± 0.06^a^	6.82 ± 0.23^a^	6.34 ± 0.07^a^
	S2	6.25 ± 0.04^a^	5.53 ± 0.23^b^	6.79 ± 0.09^a^	6.4 ± 0.14^a^
	S3	6.31 ± 0.03^a^	5.56 ± 0.09^b^	6.65 ± 0.24^a^	6.13 ± 0.02^a^
	Mean	6.35 ± 0.06	6.24 ± 0.02	6.75 ± 0.05	6.29 ± 0.01

TCC (log_10_cfumL^−1^)	S1	4.51 ± 0.22^a^	4.66 ± 0.03^a^	4.38 ± 0.03^a^	4.45 ± 0.06^a^
	S2	4.46 ± 0.17^a^	4.74 ± 0.04^a^	4.41 ± 0.04^a^	4.37 ± 0.03^a^
	S3	4.58 ± 0.10^a^	4.21 ± 0.03^a^	4.49 ± 0.02^a^	4.4 ± 0.04^a^
	Mean	4.52 ± 0.03	4.54 ± 0.02	4.43 ± 0.01	4.41 ± 0.05

Molds (log_10_cfumL^−1^)	All	ND	ND	ND	ND

*Note:* Means with different superscripts within the same column are significantly (*p* < 0.05) different.

Abbreviations: ND, not detected; S1, Sample 1; S2, Sample 2; S3, Sample 3.

**Table 6:** Physicochemical properties of the raw CM samples (mean ± std).

** **	**Sample code**	**Physicochemical properties**							
		**Fat (%)**	**Solid-not-fat (%)**	**Protein (%)**	**Lactose (%)**	**Total solid (%)**	**T. acidity (%)**	**S. gravity (%)**	**pH value**
Baka Jama	S1	5.49 ± 0.15^b^	8.39 ± 0.08^f^	3.61 ± 0.10^a^	4.61 ± 0.02^e^	13.15 ± 0.28^abcd^	0.18 ± 0.00^a^	1.027 ± 0.00^a^	6.52 ± 0.03^a^
	S2	5.76 ± 0.01^a^	8.59 ± 0.01^e^	3.52 ± 0.01^a^	4.1 ± 0.1^g^	13.15 ± 0.20^abc^	0.18 ± 0.01^a^	1.029 ± 0.00^a^	6.39 ± 0.02^ab^
	S3	5.38 ± 0.10^b^	8.78 ± 0.01^d^	3.1 ± 0.10^e^	4,81 ± 0.02^d^	12.16 ± 0.20^cd^	0.18 ± 0.00^a^	1.027 ± 0.00^a^	6.16 ± 0.15^c^

Burka Jato	S1	5.43 ± 0.0^b^	8.04 ± 0.0^h^	3.18 ± 0.00^e^	4.84 ± 0.0^c^	13.43 ± 0.0^ab^	0.17 ± 0.00^a^	1.03 ± 0.00^a^	6.38 ± 0.04^b^
	S2	5.1 ± 0.01^c^	8.88 ± 0.0^c^	3.23 ± 0.00^c^	4.87 ± 0.0^c^	13.69 ± 0.59^a^	0.16 ± 0.00^a^	1.031 ± 0.0^a^	6.19 ± 0.0^c^
	S3	4.8 ± 0.01^d^	7.13 ± 0.06^g^	2.62 ± 0.01^h^	3.94 ± 0.01^h^	12.01 ± 0.0^d^	0.16 ± 0.00^a^	1.030 ± 0.00^a^	6.18 ± 0.16^c^

Cheleleki	S1	4.14 ± 0.12^f^	9.38 ± 0.01^b^	3.43 ± 0.01^b^	5.15 ± 0.01^a^	12.63 ± 0.4^cd^	0.18 ± 0.00^a^	1.027 ± 0.00^a^	6.34 ± 0.03^b^
	S2	5.15 ± 0.10^c^	9.75 ± 0.05^a^	3.31 ± 0.01^b^	4.95 ± 0.01^b^	12.33 ± 0.57^d^	0.18 ± 0.01^a^	1.029 ± 0.00^a^	6.38 ± 0.02^ab^
	S3	3.58 ± 0.30^h^	8.25 ± 0.0^g^	2.93 ± 0.00^f^	4.41 ± 0.0^f^	11.9 ± 0.10^d^	0.18 ± 0.01^a^	1.027 ± 0.00^a^	6.42 ± 0.0^ab^

Bakanisa Kese	S1	2.8 ± 0.18^i^	8.57 ± 0.0^e^	3.14 ± 0.0^e^	4.70 ± 0.0^d^	11.59 ± 0.60^d^	0.17 ± 0.00^a^	1.03 ± 0.00^a^	6.51 ± 0.09^a^
	S2	4.58 ± 0.02^e^	7.74 ± 0.02^i^	2.8 ± 0.01^j^	4.15 ± 0.14^g^	12.13 ± 0.15^cd^	0.16 ± 0.00^a^	1.029 ± 0.00^a^	6.44 ± 0.07a^b^
	S3	3.76 ± 0.8^g^	7.03 ± 0.1^k^	2.35 ± 0.14^i^	3.33 ± 0.03^i^	11.54 ± 0.27^d^	0.16 ± 0.00^a^	1.03 ± 0.00^a^	6.44 ± 0.03^ab^

Total mean		4.66 ± 0.8	8.38 ± 0.7	3.01 ± 0.3	4.49 ± 0.5	12.48 ± 08	0.21 ± 0.14	1.029 ± 0.02	6.36 ± 0.1

*Note:* Means with different superscripts within the same row are significantly (*p* < 0.05) different.

Abbreviations: S, specific; S1, Sample 1; S2, Sample 2; S3, Sample 3; T, titratable.

## Data Availability

All the required data will be made available upon request.
